# Response to comment by Guerra et al.

**DOI:** 10.1042/BSR20181493

**Published:** 2018-10-31

**Authors:** Xinyue Yang, Yan Cheng, Guanfang Su

**Affiliations:** Department of Ophthalmology, The Second Hospital of Jilin University, Changchun 130041, China

**Keywords:** ANGPTL4, angiogenesis, inflammation, ocular diseases, vascular permeability

## Abstract

This is a response by the authors of the review article ‘A review of the multifunctionality of angiopoietin-like 4 in eye disease’ [*Biosci. Rep.* (2018) **38**, BSR20180557, https://doi.org/10.1042/BSR20180557] to the comment published in this issue by Guerra et al. [*Biosci. Rep.* (2018) **38**, BSR20180557, https://doi.org/10.1042/BSR20181462]

We would like to thank Guerra et al. [1] for their comments on our article, a review of the multifunctionality of angiopoietin-like 4 in eye disease, highlighting an important issue. In our article, we examined the importance of angiopoietin-like protein 4 (ANGPTL4) in eye diseases including sickle cell retinopathy (SCR) [2]. Jee et al. [3] studied autopsied eyes, aqueous, and vitreous samples and found that the expression of ANGPTL4 was increased in patients with SCR. We agree that the terms ‘aqueous’ and ‘*in vitro*’ were used inaccurately in our article. *In vitro* studies are performed with microorganisms, cells, or biological molecules outside their normal biological context. Whereas, *in vivo* studies test the effects of various biological entities on whole, living organisms or cells, as opposed to tissue extracts or dead organisms. For translational research purposes, *in vivo* testing is often preferred over *in vitro* testing because it provides more actionable scientific evidence. *Ex vivo* refers to experiments or measurements that are done in or on tissue from an organism in an external environment with minimal alteration of natural conditions. It may be more accurate to state that Jee et al. observed increasing expression of ANGPTL4 in autopsied eyes, aqueous, and vitreous samples of proliferative sickle cell retinopathy patients compared with controls.

We thank Guerra et al. for their consideration and offer our respects to them again.
